# Mitochondrial Dysfunction in Traumatic Brain Injury and Its Theranostic Implications

**DOI:** 10.3390/biom16060762

**Published:** 2026-05-22

**Authors:** Vratko Himic, Nana Tchantchaleishvili, Andrii Netliukh, Salvatore Chibbaro, Nikolaos Syrmos, Gianfranco K. I. Ligarotti, Lara Prisco, Mario Ganau

**Affiliations:** 1 Department of Neurological Surgery, University of Miami Miller School of Medicine, Miami, FL 33136, USA; 2 School of Medicine, BAU International University Batumi, 6010 Batumi, Georgia; 3 Department of Neurosurgery, University of Siena, 53100 Siena, Italy; 4 School of Medicine, Aristotle University of Thessaloniki, 54124 Thessaloniki, Greece; 5 Aerospace Medical Institute of Milan “A. Mosso”, Italian Air Force, 20138 Milan, Italy; 6 Nuffield Department of Clinical Neurosciences, University of Oxford, Oxford OX3 9DU, UK

**Keywords:** traumatic brain injury, mitochondrial dysfunction, neuroenergetics, oxidative stress, theranostics, neurosurgery, neuro-critical care

## Abstract

Background: Traumatic brain injury (TBI) remains a major cause of neurological morbidity and mortality. Mitochondria, being embedded as one of the key organelles disrupted after injury, play a central role in regulating neuronal metabolism, oxidative balance, and cell survival, hence the growing interest in their role after TBI. Methods: We present a narrative review of the literature on mitochondrial dysfunction after TBI to highlight the potential role in diagnosis, monitoring, prognostication and treatment strategies. Following SANRA guidelines we conducted a synthesis of 159 selected references published between 1997 and 2026, including 70 references published from 2020 onward. Results: Mitochondrial dysfunction underpins bioenergetic failure through the impairment of critical regulatory pathways, including oxidative phosphorylation, dysregulated reactive oxygen species production, and dysregulated calcium handling. These changes trigger downstream processes of oxidative damage, epigenetic and proteomic remodeling, and activation of regulated cell death pathways such as apoptosis, necroptosis, and ferroptosis in the context of an inflammatory milieu. As such, mitochondrial-derived molecules (such as mitochondrial DNA and microRNA) are emerging candidate biomarkers of TBI severity and prognosis. Additionally, therapeutic approaches under investigation include inhibition of the mitochondrial permeability transition pore, mitigation of mitochondrial oxidative stress using targeted antioxidants, restoration of NAD^+^-dependent metabolic pathways, and metabolic support through ketogenic interventions. Conclusions: Mitochondrial biology is advancing our understanding of TBI and offers a promising framework for improving its management.

## 1. Introduction

Traumatic brain injury (TBI) remains at the forefront of neurosurgical clinical care, affecting up to 69 million people annually [1]. Given such global burden, a continued improvement in the understanding of this phenomenon is needed [2].

The effects of TBI on the brain occur at both the macroscopic and microscopic strata, and TBI pathophysiology is commonly understood as the interaction between primary and secondary injury. Primary injury occurs at the moment of mechanical impact and includes tissue deformation, vascular disruption, axonal stretch or shearing, and necrotic cell death in severely damaged regions [3,4,5,6]. Secondary injury evolves over minutes to days and involves molecular mechanisms which give rise to cellular processes ultimately underpinning the medium-to-long-term sequelae. At the molecular level, these include excitotoxicity [7], oxidative stress [8], calcium dysregulation [9], neuroinflammation [10] and (epi)genomic changes [11]. At the cellular level there are further changes still: neurons die through apoptosis, necrosis and ferroptosis [12]; glia are activated and induce neuroinflammation with astrocytic scar formation and oligodendrocyte loss [13]; synapses lose function through dendritic spine loss and subsequent neurotransmission impairment [14], and impairments in neurovascular coupling occur with capillary rarefaction [15]. Ultimately, these molecular and cellular processes give rise to macroscopic changes, including in the form of diffuse axonal injury (DAI), blood–brain barrier (BBB) opening and cerebral edema [16].

Key organelles that are intimately involved in many of these processes are mitochondria. Partly due to their role in cellular functioning and signaling dynamics in various neurological conditions, their involvement in TBI has also been of particular interest [11,17,18]. Indeed, the mitochondria are thought to act as central hubs, because of their intimate regulation of excitotoxicity, calcium overload, reactive oxygen species (ROS) generation, and enzymatic degradation through caspases [19]. Mitochondria are particularly important in this secondary phase because they are central to the intrinsic apoptotic pathway. In this way, the secondary deleterious cascades that arise from mitochondrial dysfunction lead to an energy failure state that underpins the higher-order cellular and global/network changes that are seen clinically in TBI.

**Figure 1:**
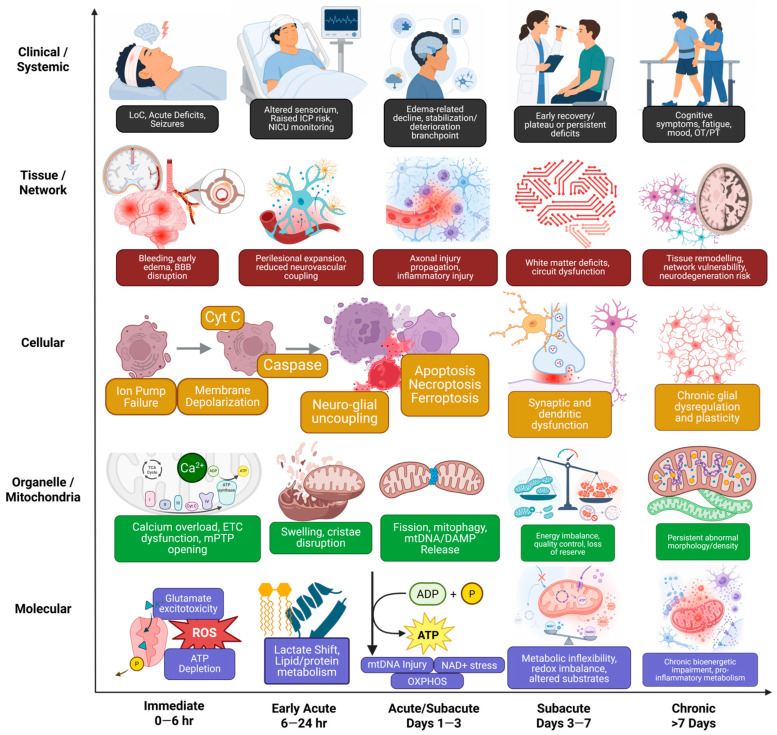
Spatiotemporal evolution of mitochondrial dysfunction after traumatic brain injury. The figure summarizes the progression of mitochondrial dysfunction after TBI across both time and biological scale. The x-axis represents time after injury, from the immediate post-traumatic period through early acute, subacute and chronic phases. The y-axis represents biological scale, ranging from molecular and biochemical events to mitochondrial, cellular, tissue/network and clinical/systemic consequences. Early events include excitotoxicity, calcium dysregulation, ATP depletion, ROS generation, ETC impairment and mPTP opening. Over subsequent hours to days, these changes evolve into oxidative stress, impaired OXPHOS, mitochondrial swelling, fission/fusion imbalance, mitophagy activation, cytochrome c release and regulated cell death. In the subacute and chronic phases, persistent mitochondrial dysfunction contributes to neuroinflammation, tissue and network disruption, altered plasticity and longer-term neurological sequelae. Abbreviations: ADP, adenosine diphosphate; ATP, adenosine triphosphate; BBB, blood–brain barrier; Ca^2+^, calcium ion; Cyt C, cytochrome c; DAMP, damage-associated molecular pattern; ETC, electron transport chain; ICP, intracranial pressure; LoC, loss of consciousness; mPTP, mitochondrial permeability transition pore; mtDNA, mitochondrial DNA; NAD^+^, nicotinamide adenine dinucleotide; NICU, neuro-intensive care unit; OT/PT, occupational therapy/physical therapy; OXPHOS, oxidative phosphorylation; P, inorganic phosphate; ROS, reactive oxygen species; TBI, traumatic brain injury; TCA cycle, tricarboxylic acid cycle.

In this narrative review we delve deeper into the vulnerability of mitochondria and their disruption in TBI. Our research question is therefore meant to evaluate the underlying biochemical and metabolic alternations which underpin these changes at the intra-cellular level. Downstream to these biochemical alterations are proteomic and epigenomic alterations which have far-reaching consequences. Secondary to these changes are the deleterious impacts which arise, including mitophagy and apoptosis (Figure 1). After covering all those processes of secondary damage, we will turn to the theranostic implications of mitochondrial disruption. This will offer the opportunity to explore the ways discoveries related to mitochondrial biology have been applied clinically to improve diagnostics (through biomarkers and imaging modalities) and to identify new therapeutic targets for TBI patients.

## 2. Methods

This article was prepared as a narrative review and was informed by principles from the Scale for the Assessment of Narrative Review Articles (SANRA). No formal risk-of-bias assessment, or quantitative synthesis was undertaken. Instead, the literature was selected to provide a mechanistic synthesis of mitochondrial dysfunction in TBI, with a particular emphasis on bioenergetic failure, oxidative stress, calcium dysregulation, proteomic and epigenomic remodeling, mitochondrial quality control, regulated cell death pathways, biomarkers, and emerging therapeutic strategies.

Multiple databases were searched throughout this narrative review, including: PubMed, Scopus, ClinicalTrials.gov, Google Scholar and Embase. Search terms were used alone and in combination, and included: “traumatic brain injury”, “mitochondrial dysfunction”, “bioenergetics”, “oxidative stress”, “mitochondrial permeability transition pore”, “mitophagy”, “apoptosis”, “ferroptosis”, “mitochondrial DNA”, “microRNA”, “biomarker”, and “theranostics”. No language restrictions were applied. The final reference list includes 159 selected sources published between 1997 and 2026, of which 70 (44.0%) were published from 2020 onward, reflecting the recent expansion of work on mitochondrial mechanisms, biomarker development, and theranostic approaches in TBI.

We also ensured that seminal works were included where they established key mechanistic concepts, while recent original experimental, translational, and clinical studies were prioritized where available and relevant to the discussion. Given the heterogeneity of the available literature, the findings were synthesized qualitatively rather than quantitatively.

## 3. Mitochondrial Role and Vulnerability in TBI

Mitochondrial dysfunction and energy deprivation in neurons and glia are a central feature of the intracellular consequences of TBI. The central role of mitochondria in neural integrity is also the reason for their vulnerability. When mitochondrial dysfunction occurs, the energy demands of the cell cannot be met and the subsequent ROS-induced damage to DNA and proteins paves the way for apoptosis [20,21]. Despite the brain’s 2% contribution to brain mass, it consumes 20% of the body’s total energy requirement, making deflections in the energy balance among neurons and glia particularly damaging.

Before we outline how mitochondria become deficient, it is important to first briefly outline their function. The role of mitochondria in metabolism lies in their role in the conversion of resource-based energy (taken from food/nutrition) into adenosine triphosphate (ATP) synthesis through oxidative phosphorylation and the final step of cellular respiration, the electron transport chain (ETC) [22]. The enzymes contained within the second inner membrane of the mitochondrion are used in key biochemical processes that allow the day-to-day functioning of each cell (including amino acid metabolism, the Krebs cycle, and fatty acid metabolism) [23]. Mitochondria have a central role in respiration and can be understood through the lens of bioenergetics [24]. Glycolysis, followed by the Krebs cycle and the ETC are the mechanisms through which mitochondria convert organic compounds to usable energy in the form of ATP [25]. This is a key area of dysfunction in TBI and is discussed further below.

Furthermore, mitochondria are not static; they are dynamic organelles that often undergo morphological changes, including fission, fusion and mitophagy [26]. Fission and fusion counterbalance each other based on the physiological and energetic needs of the cell. Fission is achieved through the generation of rings and spirals on the surface of the mitochondrion made up of dynamin-related proteins (DRPs). Fusion is achieved by mitofusin 1 and 2 (MFN1, MFN2) and optic atrophy protein 1 (OAP1) [26]. Whilst this fusion/fission interplay has typically been attributed to inheritance regulation and cellular-aging processes [27], disruption here has also been seen in TBI [28]. In addition to fission and fusion, mitophagy and biogenesis are the remaining processes that control the number of mitochondria to meet the demands of the cell; in mitophagy, mitochondria are removed through lysosomes to prevent dysfunctional mitochondria from remaining in the cytoplasm. The balance of fission and fusion directs the extent to which mitochondria are removed or generated [29,30,31].

In addition, mitochondria play a key role in cellular thermal regulation, calcium storage and transmembrane potential regulation [32,33,34,35]. The involvement of these processes makes them key in regulating autophagy, the process through which deleterious metabolic are removed; mitochondrial autophagy ensues when high levels of these products (ROS and Ca^2+^) induce the opening of the mitochondrial permeability transition pore (mPTP), affecting the integrity of the mitochondrial membrane [36]. Calcium overload, ROS production and loss of membrane potential can promote outer mitochondrial membrane permeabilization and release of cytochrome c into the cytosol. Cytochrome c then binds Apaf-1 to form the apoptosome, which activates caspase-9 and subsequently caspase-3 [37]. Recent mouse work showed that mild TBI induces hippocampal mitochondrial calcium overload, reduced mitochondrial membrane potential and compensatory upregulation of the mitochondrial Na^+^/Ca^2+^ exchanger NCLX, reinforcing calcium dysregulation as an early driver of mitochondrial vulnerability after injury [38]. In parallel, p53 can contribute to mitochondrial apoptosis through transcription-dependent effects on pro-apoptotic genes and through non-transcriptional interactions with Bcl-2 family proteins at the mitochondrial membrane [39,40]. These pathways are particularly relevant after TBI, where mitochondrial permeabilization links acute metabolic stress to delayed apoptotic cell death.

The same physiological properties that make mitochondria essential to neuronal and glial function also make them particularly vulnerable after TBI. Neurons have high ATP requirements, alongside a strong dependence on mitochondrial oxidative phosphorylation for maintenance of synaptic transmission and ion gradients. In glial cells, mitochondrial dysfunction can alter inflammatory activation, astrocytic metabolic support, and neuron–glia signaling [41]. In addition, blast-induced TBI has been shown to disrupt astrocytic mitochondrial dynamics, supporting the view that post-traumatic mitochondrial dysfunction is not restricted to neurons [42,43]. Recent experimental work using human three-dimensional triculture brain tissue models has shown that post-contusion injury induces mitochondrial dysregulation across neurons, astrocytes and microglia, contributing to secondary neurodegeneration [44].

**Figure 2:**
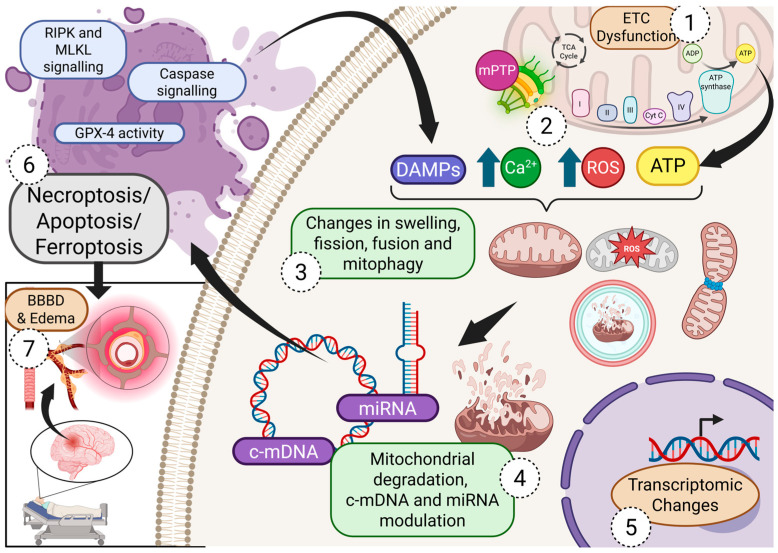
Schematic summarizing the consequences of mitochondrial dysfunction in TBI pathophysiology. 1: ETC dysfunction within the mitochondrial organelle. 2: The mPTP is disrupted and increased amounts of toxic calcium and ROS build-up, further exacerbating mitochondrial dysfunction. 3: Mitochondria swell and experience changes to the regulation of fusion, fission and mitophagy. 4: Some mitochondria undergo failure and c-mDNA is released. Alongside miRNAs these lead to downstream changes at the level of (5) transcription as well as post-transcriptomic changes which are reflected in both transcriptomic and proteomic studies. 6: Cells undergo cell death via various distinct mechanisms and pathways; they then release DAMPs which further exacerbate the pro-inflammatory cytotoxic milieu and extracellular matrix. This cycle can repeat and affect a progressively larger area. 7: When repeated on a large area/scale, there is blood–brain barrier disruption as well as cerebral edema. Abbreviations: ETC—electron transport chain; TCA—tricarboxylic acid/Krebs cycle; mPTP—mitochondrial permeability transition pore; ROS—reactive oxygen species; DAMPs—damage associated molecular patterns; miRNA—micro RNA; c-mDNA—circulating mitochondrial DNA; BBBD—blood–brain barrier disruption; RIPK—Receptor Interacting Protein Kinases; glutathione peroxidase 4 (GPX4); ATP—adenosine triphosphate.

Taken together, Figure 2 summarizes the major downstream consequences of mitochondrial dysfunction after TBI. These include disruption of the ETC, excessive calcium and ROS accumulation, mPTP opening, mitochondrial swelling, altered fission/fusion and mitophagy, release of mitochondrial-derived damage-associated molecular patterns, transcriptional and post-transcriptional changes, and activation of regulated cell death pathways. Although these processes are presented schematically, they should be understood as interconnected rather than linear, with mitochondrial damage amplifying both local cellular injury and larger tissue-level consequences such as blood–brain barrier disruption and cerebral edema.

These early disturbances in mitochondrial structure and signaling provide the basis for the more specific metabolic and energetic abnormalities discussed below.

## 4. Metabolic and Energetic Disruption

Moving forward, we can discuss the concepts of energetic failure in TBI. Energetic failure is one of the most reproducible biochemical consequences of TBI. Stress signals such as the release of excitatory neurotransmitters like glutamate, contribute to a multi-factorial process that underpins mitochondrial changes in energy processing [45]. Early human cortical studies were able to demonstrate that reduced ATP levels are present in TBI-simulated tissue even when that tissue is not yet ischemic, indicating an intrinsic role of mitochondrial dysfunction rather than purely perfusion-related compromise [46]. Here, in a subset of their study cohort, Verweij and colleagues showed that mitochondria isolated from surgically removed brain tissue (in those with intracranial hypertension secondary to TBI) displayed significant metabolic changes compared to controls.

Further experimental work by Pandya and colleagues used young adult male rats to show that when treated with mitochondrial uncouplers, the models had both improved cortical tissue sparing and behavioral outcomes, which they attributed to the reduction in mitochondrial Ca^2+^ uptake and ROS production [47]. Homeostasis of calcium flux is a key role of mitochondria, particularly in their role of sequestering Ca^2+^ in times of high cytosolic levels. When the immunosuppressant cyclosporin A is administered peripherally, it reduces CNS dysfunction in animal models of TBI in a dose-dependent way, achieving its effect by blocking the usual functioning of the mitochondrial permeability transition pore which would typically extrude high mitochondrial Ca^2+^ [48].

In the context of metabolic alterations, oxidative stress is closely coupled with these bioenergetic disturbances. Oxidative stress occurs when ROS are elevated and damage DNA, proteins and lipids [49]. A key mouse study by Singh and colleagues demonstrated that there are serial changes in the mitochondria that occur in post-traumatic states, and they evolve with an initial uncoupling of the ETC followed by eventual destabilization of respiratory control. Mitochondria are then seen to be swollen, with disrupted cristae and ruptured outer membranes [50]. They showed that these changes are not only extensive, but that they are mediated by reactive species and change over time, suggesting that there is a time-window during which future therapeutic approaches should take place, particularly by the 3 h mark [51].

Mitochondrial energy failure after TBI therefore is not simply defined by ROS production and calcium overload alone. Damage to respiratory chain complexes reduces oxidative phosphorylation efficiency, promotes electron leakage and uncouples oxygen consumption from ATP synthesis [52]. The resulting ATP deficit impairs Na^+^/K^+^-ATPase and Ca^2+^-ATPase activity, worsening membrane depolarization, glutamate release and excitotoxic signaling. In parallel, injured tissue may shift from a state of oxidative phosphorylation toward glycolytic metabolism as an adaptive response to impaired mitochondrial respiration [53]. Although this may transiently support ATP generation, impaired pyruvate oxidation can lead to lactate accumulation, increased lactate/pyruvate ratios and tissue acidosis, further aggravating cellular injury [54]. This can therefore be appreciated as a reprogramming of metabolic pathways in a state of energy failure.

In this context, metabolic inflexibility has emerged as an additional feature of mitochondrial dysfunction after TBI. Early experimental work by Prins and colleagues revealed that injured tissue increases ketone body uptake and oxidation, suggesting that ketone metabolism can partially bypass glycolytic impairment and support mitochondrial oxidative phosphorylation. They showed that cerebral uptake and oxidation of beta-hydroxybutyrate was seen in a TBI model and, furthermore, ketone supplementation was able to restore reduced cortical ATP levels, indicating that ketone bodies can support mitochondrial energy metabolism after injury [55,56]. Because ketone bodies enter the Krebs cycle downstream of glycolysis, they seem to provide an alternative energy substrate when glucose-centered metabolism is impaired. Beta-hydroxybutyrate has been linked with improving general efficiency of respiratory processes and the reduction in oxidative stress during times of metabolic disturbance, as in the case of the ketogenic diet [57].

More recently, translational work has explored ketogenic metabolic therapy in patients with severe TBI. A phase I trial demonstrated that therapeutic ketosis can be safely achieved in the neurocritical care setting using a ketogenic diet protocol [58]. In this trial, achieving ketosis (as measured by beta-hydroxybutyrate levels) was the primary outcome. Although only 10 patients were assessed, they were able to achieve ketosis in 8 of 10 patients, although two developed hypertriglyceridemia and one developed hypoglycemia, paving the way for dose–response trials in larger cohorts [58,59]. There is also evidence that repeat TBI is additive in its damage to metabolic processes; single and repeated mild TBI in male rats was shown to produce acute changes in neurometabolites, lipids and mitochondrial function, supporting the concept that post-traumatic metabolic dysfunction extends beyond ATP depletion alone [60].

These metabolic disturbances do not remain confined to acute energy failure but also shape longer-lasting proteomic, transcriptomic and epigenomic responses.

## 5. Proteomic and Epigenomic Alterations

After having discussed the cellular and compartmental changes that occur during the energetic disruption that mitochondria partake in during TBI, we will now outline how this dysfunction is further reinforced by widespread proteomic and epigenetic remodeling that extend beyond acute metabolic disturbances. The shift from bioenergetic studies to proteomic and transcriptomic studies reveals the efforts to try to find druggable targets to enhance neuroprotection and prevent permanent neurological damage in patients hospitalized with TBI.

Early rodent work demonstrated that TBI induces oxidative modification and functional impairment of mitochondrial proteins. Opii and colleagues used a controlled cortical impact rat model with proteomic analysis to show that several mitochondrial fraction proteins were oxidatively modified, such as cytochrome C oxidase, enolase-1, pyruvate dehydrogenase, fumarate hydratase 1, ATP synthase, and prohibitin, among others, further giving credence to the early ideas that mitochondrial bioenergetic breakdown occurs as a consequence of widespread protein modifications in response to TBI [50,61].

Epigenetic regulation has also emerged as an important modulator of mitochondrial responses to TBI. Balasubramanian and colleagues showed that in the hippocampi of rats that underwent repeated mild TBI, there was hypermethylation of mitochondrial transcription factor A (TFAM) as well as mitochondrial DNA promoters HSP1 and HSP2, showing decreases in rRNA, mRNA, as well as protein levels. They were able to reverse this by treating with methionine and restoring the hypermethylated state back to normal levels [62]. In parallel, nuclear epigenetic modifications after TBI, including changes in global methylation and chromatin regulation, have also been recognized as contributors to sustained transcriptomic remodeling, with suggestions that when epigenomic changes associated with TBI persist, punishing pathway changes can predispose and facilitate future increased incidence of various neurodegenerative diseases [63].

More recent transcriptomic and mechanistic studies have clarified these changes at the epigenetic level. Dong and colleagues sequenced brain tissue samples from TBI patients and compared them to controls; they found that genes responsible for mitochondrial oxidative phosphorylation were downregulated whilst pathways involved in neurodegeneration were upregulated [53]. In addition, they showed that pro-inflammatory signaling pathways were upregulated, a finding which they demonstrated in vitro and showed that a peroxisome proliferator-activated receptor gamma coactivator 1-alpha (PGC-1α) activator was able to rescue the oxidative stress and apoptosis induced by TNF-α exposure. Further work on the PGC-1α pathway has also suggested a key regulatory involvement in mitochondrial processing of oxidative stress. Natural anti-inflammatory agents such as quercetin and histone deacetylases such as sirtuin 1 (of the SIRT family) have both been shown to play a key protective role in the secondary injury that occurs after TBI [64,65]. Among these, many other mitochondrial agents have been investigated through their targeting of mitochondria in TBI. Furthermore, although direct in vivo evidence of NAD^+^ depletion altering the aforementioned SIRT1 activity after TBI remains limited, recent mechanistic reviews link NAD^+^-dependent SIRT1 deacetylase pathways with PGC-1α/TFAM regulatory networks that govern mitochondrial biogenesis and chromatin accessibility in response to cellular stress [52].

Post-transcriptional regulation by microRNAs (miRNAs) adds another layer of control as well as another potential are exploitation in both the deleterious as well as the therapeutic sense. Mitochondrial miRNAs show a dynamic and coordinated expression pattern in response to TBI [66,67]. Several miRNAs have been shown to be consistently dysregulated following TBI and have been observed in mitochondria-associated fractions. For example, miR-21 is upregulated in rodent hippocampal tissue after TBI, whereas miR-155 and miR-223 are upregulated in cortical tissue; the latter two are involved in immune and hematopoietic responses [68,69]. Various injury-associated miRNAs are elevated in damaged cortex and correlate with secondary injury signaling [70]. Recent work has also implicated circular RNA regulation in post-traumatic mitochondrial injury, with circIgfbp2 inhibition alleviating mitochondrial dysfunction and oxidative stress-induced synaptic impairment through the miR-370-3p/BACH1/HO-1 axis [71].

Collectively, these findings suggest that mitochondrial dysfunction after TBI is not solely the consequence of acute biochemical stress but is sustained by coordinated alterations in epigenetic and post-transcriptional regulatory mechanisms. These regulatory changes help explain how acute mitochondrial injury can evolve into sustained cellular vulnerability and regulated cell death.

## 6. Mitochondria and Cell Death Pathways

After having discussed the relevance of mitochondria in normal cellular function and exploring the ways these functions are affected in TBI, including at the proteomic and epigenetic levels, we now turn to the culmination of these intracellular changes. We discuss the mechanisms of cell death that ultimately underpin both the short as well as long-term consequences of TBI.

### 6.1. Apoptosis

As we have outlined before, mitochondria act as central regulators of neuronal survival and death after TBI. One of the earliest mechanisms described in this process involves changes in permeability of the mitochondrial outer membrane and the subsequent release of cytochrome c from the intermembrane space into the cytosol [72,73]. This then activates the intrinsic apoptotic cascade through caspase-9 and caspase-3 signaling [74,75]. These rapid fluxes of cytochrome c translocation and caspase activation occur within hours of the initial insult [72,73]. These apoptotic processes can arise secondary to the aforementioned upstream mitochondrial stressors (including calcium overload and oxidative stress). The apoptosis-regulator gene *bcl-2* was shown in a rodent model to play a central role in regulation of neuronal cell death after TBI in a seminal paper by Clark and colleagues [76]. In addition, previously described factors such as p53 and E2F1 provide additional links between mitochondrial dysfunction and delayed cell death. p53 can promote mitochondrial outer membrane permeabilization through effects on Bax/Bak signaling and inhibition of anti-apoptotic Bcl-2 family proteins [39]. Recent TBI work has implicated E2F1-p53 signaling in neuronal injury, while E2F1/CDK5/Drp1 signaling has been linked to microglial mitochondrial dysfunction and neuroinflammation [40].

### 6.2. Necroptosis

In addition to classical apoptosis, more regulated necrotic pathways have received increased attention in the context of TBI. Necroptosis is mediated by the receptor-interacting protein kinases RIPK1 and RIPK3 and culminates in the phosphorylation of a mixed lineage kinase domain-like protein (MLKL), leading to membrane disruption and inflammatory cell death [77,78]. Cho and colleagues then reported on the role of RIP3 as a key activator of programmed necrosis [77], work which was expanded later to introduce MLKL as an interactive target; when MLKL was knocked down in vitro it halted the necroptosis process [78]. This necroptosis has then been implicated in TBI [79]; for example, the aforementioned necrostatin-1 was shown to reduce neuronal loss and tissue damage in a controlled cortical impact mouse model. More recent studies have confirmed that increased RIPK3 and MLKL activation follow, partly through reducing neutrophil influx and microglial activation [80]. Further studies strengthened evidence of necroptosis and its role in contributing to the pro-inflammatory milieu of TBI [81,82].

### 6.3. Ferroptosis

In addition to classical apoptosis and now necroptosis, ferroptosis has emerged as another important mechanism linking mitochondrial dysfunction to neuronal death in TBI. This iron-dependent form of regulated cell death is characterized by lipid peroxidation, glutathione depletion and impaired glutathione peroxidase-4 (GPX4) activity [83,84]. Evidence for ferroptotic signaling in TBI was demonstrated by Kenny and colleagues, who showed both in vitro and in vivo that markers of ferroptosis are increased post-TBI (such as oxidized phosphatidylethanolamine and 15-lipoxygenase as well glutathione depletion); administration of a 12/15-lipoxygenase inhibitor baicalein was able to improve outcomes in mice [85]. Subsequent work has supported a role for iron-driven oxidative damage and mitochondrial lipid peroxidation in the amplification of neuronal injury during the secondary phase of TBI [86,87,88].

### 6.4. Mitophagy and Mitochondrial Quality Control

Mitochondrial quality control processes also influence neuronal fate after injury. Mitophagy, which is the selective autophagic removal of damaged mitochondria, is activated following TBI and may initially serve a protective role by limiting the accumulation of dysfunctional mitochondria [52,89]. Activated as a response to the canonical stimuli of oxidative stress, mitochondrial transmembrane flux alterations and a pro-inflammatory milieu, it helps to mediate and prevent an overly inflammatory response [90,91]. However, sustained or excessive mitophagy may actually act to reduce mitochondrial mass (and hence their protective respiratory capacity), exacerbating bioenergetic failure and promoting neuronal vulnerability [92,93,94,95]. This dual role further underscores the temporal complexity of mitochondrial quality control pathways, whilst it also increases the list of potential druggable targets.

More recent mitophagy work has taken these findings further. Zhang and colleagues showed that human umbilical cord mesenchymal stem cell-derived exosomes can suppress programmed cell death after TBI through PINK1/Parkin-mediated mitophagy, adding a translational extracellular vesicle angle to mitochondrial quality control [96]. Similarly, astrocyte-derived exosomal GJA1-20k (a truncated isoform of connexin 43) was found to attenuate the effects of TBI by targeting Pink1-mediated mitophagy [97]. Lastly, copper accumulation can aggravate post-TBI synaptic damage by downregulating BNIP3-mediated mitophagy, implicating impaired mitochondrial clearance in post-traumatic synaptic dysfunction [98].

### 6.5. Long-Term Consequences of Mitochondrial Dysfunction

Whilst much of the pathological processes we have discussed have been in the acute and subacute phase, mitochondrial dysfunction can likewise contribute to chronic post-traumatic pathology. Persistent oxidative phosphorylation impairment, mitochondrial fragmentation and redox imbalance can maintain neuroinflammatory signaling long after the acute injury [52]. Recent human 3D triculture modeling suggests that mitochondrial dysregulation after contusion injury contributes to secondary neurodegeneration progression, supporting the concept that mitochondrial injury may bridge acute trauma and chronic cellular degeneration [44]. These mechanisms are relevant to the long-term clinical phenotype of TBI, including cognitive impairment, fatigue, mood disturbance and increased vulnerability to neurodegenerative processes [99,100].

### 6.6. Temporal Distribution of Mitochondrial Dysfunction in TBI

Together, these mechanisms illustrate how mitochondrial dysfunction functions as a central hub integrating metabolic failure, oxidative stress and regulated cell death pathways, ultimately contributing to neuronal loss, axonal injury and chronic neurodegenerative processes that are the hallmark of the long-term sequelae of TBI. Although this review is organized mechanistically rather than chronologically, many mitochondrial processes after TBI are time-dependent. Table 1 therefore provides a temporal view linking mitochondrial events to clinical correlates. The same pathways that mediate mitochondrial injury and cell death also provide potential therapeutic targets and biomarker candidates, forming the basis of the theranostic approaches discussed below.

## 7. Theranostic Implications

After having discussed the central role of mitochondria in TBI-induced energetic failure, oxidative stress and cell death, we will close with an outline of how this mechanistic knowledge can be exploited through the lens of theranostics; many of these features of mitochondrial biology can be targeted as both biomarkers of TBI as well as therapeutic interventions for it.

Both preclinical and subsequent clinical work over the past two decades has shown that several key elements of mitochondrial dysfunction that occur as a consequence of TBI (including permeability transition, oxidative damage, impaired bioenergetics and defective mitochondrial quality control) may be pharmacologically modifiable [52,101,102,103]. To summarize the translational relevance of mitochondrial biology in TBI, Table 2 groups the principal therapeutic and biomarker targets according to the mitochondrial process involved, the pathological sequelae post-injury, as well the current maturity of the evidence base.

### 7.1. mPTP and Mitochondrial Permeability

One of the most extensively studied targets has been the mitochondrial permeability transition pore (mPTP). Excessive mitochondrial calcium loading following TBI promotes the opening of the mPTP, resulting in the loss of membrane potential, mitochondrial swelling and release of pro-apoptotic factors. Pharmacological inhibition of this process using cyclosporine A has demonstrated neuroprotective effects in several experimental models of TBI studied in the 2000s by preserving mitochondrial respiration and reducing neuronal loss [104,105,106]. Early translational studies subsequently evaluated cyclosporine-based therapies in patients with severe TBI, including the development of the NeuroSTAT^®^ formulation designed to optimize central nervous system penetration; this was shown to have positive effects on injury biomarkers in a phase Ib/IIa trial (NCT01825044) [107,108,109].

### 7.2. Mitochondrial Oxidative Stress

A second therapeutic strategy focuses on the mitigation of mitochondrial oxidative stress that is generated in the TBI setting. Following injury, damaged mitochondria generate excessive ROS, contributing to lipid peroxidation, protein oxidation and mitochondrial DNA damage. The development of antioxidant compounds that specifically neutralize ROS has been at the center of this [110,111]. For example, elamipretide (a peptide antioxidant) can stabilize the ETC’s function by binding in the inner mitochondrial membrane, relieving the pressure placed on the mitochondria by the ROS through CD36 down-regulation as well as restoration of SIRT1 expression and upregulation of PGC-1α [112,113,114]. Similarly, mitochondria-targeted quinone derivatives such as mitoquinone (MitoQ) have been shown to reduce mitochondrial oxidative stress and improve behavioral and cellular outcomes in rodent models of repetitive or moderate TBI [41,115], a process which is partly driven via changes in the Nrf2-ARE pathway [116]. Other examples include plastoquinone (SkQ1) [117] and ederavone [118]. Additionally, fibroblast growth factor 21 (FGF21) was shown to improve neuronal survival after TBI by maintaining mitochondrial redox homeostasis through SLC25A39-dependent mitochondrial glutathione transport, offering a newer redox-based therapeutic target [119].

### 7.3. Bioenergetic Restoration and Metabolic Support

Restoration of mitochondrial bioenergetic capacity (which shows signs of failure in the TBI context) has also emerged as a promising therapeutic approach. Because mitochondrial respiration depends heavily on the availably of cellular NAD^+^, efforts aimed at restoring the reserve of NAD^+^ could improve the extent of neural injury by supporting mitochondrial function and blocking strongly pro-inflammatory pathways (achieving this through changes to oxidative phosphorylation and activation of sirtuin-dependent pathways) [120,121]. In a similar vein, metabolic therapies designed to support mitochondrial substrate utilization have also received increasing attention. Ketogenic interventions and exogenous ketone supplementation (for example with β-hydroxybutyrate and acetoacetate) can provide an alternative fuel source capable of bypassing impaired glycolytic pathways and improving mitochondrial energy production after TBI [122,123,124]. Although many of these interventions are being scrutinized in the context of TBI in this article, it is pertinent to mention that many of these interventions have been or are being investigated in the context of neurodegenerative and ischemic cerebral conditions as many share similar mechanistic pathways with the consequences of TBI on the brain, and therefore can inform management in intensive care setting [125,126].

### 7.4. Mitochondrial Quality Control

Moving forward, alternative interventional approaches involve the targeting of mitochondrial quality control pathways to maintain mitochondrial integrity (these include mitochondrial fission, fusion and mitophagy). Dysregulation of these processes, particularly as it pertains to excessive mitochondrial fission, has been seen as a facilitator of TBI-induced neural damage. For example, inhibition of a key fission mediator called dynamin-related protein-1 (Drp1) with the small molecule inhibitor Mdivi-1 has been shown to preserve mitochondrial morphology, reduce neuronal apoptosis and improve functional outcomes in experimental models of TBI [127,128]. When pathological mitochondrial fission is blocked in its early stages, the energetic failure and chronic neurocognitive degeneration which results can be arrested. This can be achieved by pharmacologically preventing mitochondrial fission protein (Fis1) from binding to Drp1 [129]. In addition to balancing fission and fusion processes, targeting the autophagy and mitophagy pathways has also been explored as potential therapeutic strategy. Rapamycin can limit secondary injury cascades after TBI by improving the clearance of damaged mitochondrial, for example [52,130]. By activating mTOR, rapamycin inhibits the NLRP3 inflammasome and shows synergy with MCC950 (an NLRP3 selective inhibitor) [131,132,133,134].

Further recent preclinical work has expanded the mitophagy-theranostics angle, showing that allicin and D-mannose can reduce post-traumatic neuroinflammation through mitophagy-linked pathways, including PKC-δ-mediated mitophagy and AMPK-mitophagy/NLRP3 signaling [135]. Interesting work has also been done to see if transplantation of mitochondria using astrocyte-derived mitochondria could become a strategy to mitigate mitochondrial dysfunction and inflammation after TBI, although this work remains preclinical for now [136]. Melatonin has also been studied as a neuroprotective agent after TBI, with whole-transcriptome sequencing in a mouse model showing broad regulation of coding and non-coding RNA networks after treatment [137]. Whilst these results were not confined to mitochondrial biology, they give credence to the idea that recovery after TBI involves coordinated molecular remodeling, bridging mitochondrial quality control processes including fission, fusion and mitophagy.

### 7.5. Mitochondrial Biomarkers and Diagnostic Markers

In addition to therapeutic targeting, mitochondrial components themselves are increasingly being investigated as biomarkers of injury severity and outcome [138]. Mitochondrial DNA (mtDNA) released from damaged cells can act as damage-associated molecular patterns (DAMPs) that activate innate immune signaling pathways and contribute to systemic inflammation after trauma (hence the benefits of rapid and efficient clearing of mitochondria as a potential therapeutic option) [139]. Elevated circulating mtDNA levels can correlate with injury severity and patient outcomes [140,141,142], and microRNAs that are involved in mitochondrial signaling in inflammatory and regulatory pathways have also shown promise as potential biomarkers [143,144]. These can be detected in both CSF, saliva, and peripheral blood (allowing for easier, liquid biopsy monitoring) and have been used in prognostication, including through novel nanotech-based platforms [145,146,147,148].

Beyond prognostication, mitochondrial-associated markers are increasingly being explored for diagnostic classification after TBI. Cell-free mitochondrial DNA has been detected in serum and CSF after acute brain injury, with CSF levels correlating with clinical severity and IL-6-mediated inflammatory responses [149]. Extracellular vesicle-associated mitochondrial DNA has also been proposed as a diagnostic marker, with recent experimental work showing that EV-associated mtDNA, particularly when combined with serum amyloid A, can distinguish TBI from control states in a mouse model [150]. Furthermore, circulating microRNAs could provide a complementary diagnostic approach because they are stable in biofluids and may reflect injury-related mitochondrial, inflammatory and neurodegenerative pathways [151].

### 7.6. Limitations

This narrative review has some limitations that are worth considering: (1) the role of mitochondrial disruption may influence the clinical course in the extreme decades of life where the metabolic requirement may differ; for this, the role as biomarker in children and older adults (the so-called silver trauma occurring in the elderly) remains a point for future research [152,153,154,155]; (2) the differences could also be sex-specific, with severe TBI in mice producing sex-specific mitochondrial dysfunction [156]; (3) the findings reported in this narrative review may underplay the wide heterogeneity of TBI, ranging from concussion symptoms to severe compromise of wakefulness and awareness [157,158,159]; therefore, we advise caution in generalizing the weight of mitochondrial disruption to the full spectrum of clinical presentations; (4) since much of the research on novel therapeutic targets described in this review is in its early stages and is heterogenous in nature, we suggest the reader to refer to the ClincalTrials.gov pages related to the trials mentioned for further updates that will become available in the future; (5) finally, although mitochondrial biomarkers such as circulating mtDNA and microRNAs are promising, standardized sampling windows, assay platforms and validation cohorts are still needed before routine clinical implementation.

## 8. Conclusions and Future Directions

The mitochondria play a central role in TBI. Their key role in cellular functioning is precisely what makes them so susceptible to the demands put on neural tissue in the context of traumatic stress. We now know that it is not only a target of these deleterious processes, but it can also play a role as a central mediator of secondary injury. Over the past two decades, both basic science studies and human trials have synergized to uncover multiple facets of mitochondrial dysfunction, including the following: permeability transition, oxidative stress, impaired bioenergetics and defective quality control. Fortunately, significant work has been undertaken and is currently underway to see how these pathways can be targeted pharmacologically, while mitochondrial-derived molecules such as circulating mtDNA and regulatory microRNAs offer potential biomarkers of injury severity and recovery. Although many of these strategies remain at the preclinical or early translational stage, the convergence of therapeutic targeting and biomarker discovery around mitochondrial pathways provides a framework for more precise monitoring and individualized intervention following TBI, and intensive care units worldwide are bringing the findings of these studies from the bench to the bedside at an impressive pace. Continued integration of mechanistic insights into clinical biomarker development will further strengthen the case for translating mitochondrial biology into effective theranostic strategies in the management of TBI.

## Figures and Tables

**Table 1:** Timeline summary of mitochondrial events after TBI and their clinical correlates. The table outlines representative mitochondrial processes across acute, subacute and chronic phases of injury and relates these mechanisms to common clinical features. Please note that the timing of these events is approximate and may vary according to injury severity, age, comorbidity and experimental or clinical context.

Time Post-TBI	Mitochondrial Events/Processes	Mechanistic Notes/Implications	Clinical Correlates/Symptoms
**Immediate/Early (0–12 h)**	-Acute calcium influx into mitochondria -mPTP opening in some neurons -Initial ROS burst -Altered mitochondrial membrane potential	Triggers early neuronal apoptosis and necrosis; sets the stage for oxidative damage; early energy failure can impair ion homeostasis	Loss of consciousness, acute neurological deficits, confusion, headache, possible seizures
**Early (12–24 h)**	-Continued ROS production -Initial fragmentation (fission) dominates over fusion -Mitophagy activated in stressed but salvageable mitochondria	Attempted removal of damaged mitochondria; imbalance in fission/fusion contributes to bioenergetic deficits; oxidative stress peaks	Persistent confusion, disorientation, worsening headache, nausea/vomiting, early signs of edema
**Intermediate (24–72 h)**	-Persistent mitochondrial dysfunction -Increased fission, reduced fusion -Sustained ROS and lipid peroxidation -Mitophagy may be overwhelmed	Energy deficit continues; apoptosis pathways increasingly engaged; oxidative stress contributes to secondary injury	Progressive neurological deficits, fluctuating consciousness, early cognitive impairment, risk of post-traumatic seizures, signs of raised ICP
**Subacute/Late (3–7 days)**	-Gradual recovery in surviving neurons -Possible compensatory fusion and biogenesis -Continued mitophagy and clearance of severely damaged mitochondria -Persistent oxidative stress in vulnerable regions	Mitochondrial recovery or cell death depends on injury severity; potential window for therapeutic targeting (e.g., antioxidants, mPTP inhibitors)	Improvement or plateau in consciousness; persistent cognitive deficits; mood changes; motor deficits may become evident; risk of secondary complications (infection, edema)
**Chronic (>7 days)**	-Long-term changes in mitochondrial density and morphology -Altered metabolic profiles in surviving neurons -Possible persistent mitophagy and low-level ROS	Contributes to chronic neurodegeneration and functional deficits; may underlie post-TBI cognitive and behavioral impairments	Persistent cognitive deficits, fatigue, behavioral changes, memory problems, chronic headache, risk of post-traumatic epilepsy

mPTP—mitochondrial permeability transition pore; ROS—reactive oxygen species; ICP—intracranial pressure; TBI—traumatic brain injury.

**Table 2:** Mitochondrial processes as therapeutic and biomarker targets in TBI. Table summarizing the major mitochondrial domains implicated in TBI pathophysiology and their potential theranostic relevance. Therapeutic strategies include modulation of mitochondrial permeability, oxidative stress, bioenergetic failure, substrate utilization, and mitochondrial quality control. Biomarker approaches include circulating cell-free mitochondrial DNA, extracellular vesicle-associated mitochondrial DNA, microRNA panels, and multi-omic mitochondrial signatures. The translational maturity of these approaches varies substantially: several interventions have strong preclinical support, some have entered early clinical evaluation, and most biomarker strategies require larger validation cohorts and standardized sampling protocols before routine clinical use.

Domain	Relevance to TBI Pathophysiology	Theranostic Examples	Current Evidence and Translational Status
**Mitochondrial permeability and calcium handling**	Calcium overload promotes mitochondrial membrane depolarization, mPTP opening, swelling, respiratory failure, cytochrome c release, and intrinsic apoptosis.	Cyclosporine A; NeuroSTAT^®^; experimental calcium-handling modulators.	Cyclosporine A has preclinical evidence in TBI through mPTP inhibition and preservation of mitochondrial energetics. NeuroSTAT^®^ has been evaluated in early-phase severe TBI studies, with evidence of CNS penetration and biomarker effects. Direct mitochondrial calcium-targeted therapies remain preclinical.
**Mitochondrial oxidative stress**	Damaged mitochondria generate excess ROS, contributing to lipid peroxidation, protein oxidation, mtDNA injury, and further impairment of the electron transport chain.	MitoQ; SS-31/elamipretide; edaravone; plastoquinone derivatives such as SkQ1/SkQR1.	MitoQ and SS-31 have direct preclinical TBI evidence. Edaravone has supportive rodent TBI data through antioxidant/Nrf2-related mechanisms. Plastoquinone derivatives have stronger evidence in related acute CNS injury models, with less direct validation in TBI.
**Bioenergetic failure and substrate metabolism**	ETC dysfunction and impaired oxidative phosphorylation reduce ATP production, worsen ion pump failure, promote depolarization, and aggravate excitotoxicity. Compensatory metabolic changes may include glycolytic shift, lactate accumulation, and impaired substrate flexibility.	PGC-1α activation; NAD^+^ precursor strategies; ketogenic diet; β-hydroxybutyrate or acetoacetate supplementation.	OXPHOS impairment and inflammatory suppression of mitochondrial metabolism are supported by experimental and transcriptomic TBI studies. Ketogenic therapy has early human feasibility data in severe TBI. NAD^+^ restoration is mechanistically plausible and supported by broader neuro-metabolic literature, but remains less directly validated in TBI.
**Mitochondrial dynamics and quality control**	Imbalance in fission, fusion, and mitophagy contributes to mitochondrial fragmentation, reduced respiratory capacity, persistent ROS production, inflammasome activation, and delayed neuronal vulnerability.	Mdivi-1; Fis1–Drp1 interaction blockade; rapamycin; melatonin; PINK1/Parkin pathway modulation; NLRP3 inhibition.	Drp1/fission inhibition is supported by preclinical TBI studies. Rapamycin should be described as an mTOR inhibitor/modulator that can influence autophagy and mitophagy, not as an mTOR activator. Most approaches remain preclinical and timing-dependent.
**Mitochondrial-derived biomarkers**	Damaged mitochondria release mtDNA and other mitochondrial danger signals that can activate innate immune pathways and may reflect injury severity. Mitochondrial-associated regulatory RNAs may reflect inflammatory and recovery pathways.	Serum/CSF cell-free mtDNA; extracellular vesicle-associated mtDNA; blood, CSF, or saliva microRNA panels.	Cell-free mtDNA has human acute brain injury evidence and may correlate with severity and inflammatory response. EV-associated mtDNA remains preclinical for TBI diagnosis. Biofluid microRNA panels show diagnostic and prognostic promise but require assay standardization and larger validation cohorts.
**Integrated mitochondrial signatures**	Persistent mitochondrial dysfunction may connect acute secondary injury with chronic neuroinflammation, neurodegeneration, and persistent cognitive or behavioral symptoms.	Multi-omic profiling, including transcriptomics, proteomics, metabolomics, and integrated biomarker panels.	An emerging translational framework for patient stratification and mechanistic monitoring, but not yet routine clinical practice.

Abbreviations: TBI, traumatic brain injury; mPTP, mitochondrial permeability transition pore; ROS, reactive oxygen species; mtDNA, mitochondrial DNA; ETC, electron transport chain; OXPHOS, oxidative phosphorylation; ATP, adenosine triphosphate; CNS, central nervous system; Nrf2, nuclear factor erythroid 2-related factor 2; NAD^+^, nicotinamide adenine dinucleotide; PGC-1α, peroxisome proliferator-activated receptor gamma coactivator 1-alpha; Drp1, dynamin-related protein 1; Fis1, mitochondrial fission 1 protein; PINK1, PTEN-induced kinase 1; Parkin, E3 ubiquitin ligase Parkin; EV, extracellular vesicle; CSF, cerebrospinal fluid.

## Data Availability

No new data were created or analyzed in this study.
